# Guiding-sheath-assisted rescue deployment of a fully covered biliary metal stent after release failure during endoscopic retrograde cholangiopancreatography

**DOI:** 10.1055/a-2825-8500

**Published:** 2026-03-24

**Authors:** Koichi Soga, Ou Takagi, Haruka Kato, Masaru Kuwada, Ryosaku Shirahashi, Ikuhiro Kobori, Masaya Tamano

**Affiliations:** 1 26263 Department of Gastroenterology, Dokkyo Medical University Saitama Medical Center, Koshigaya, Japan

**Fig. 1:**
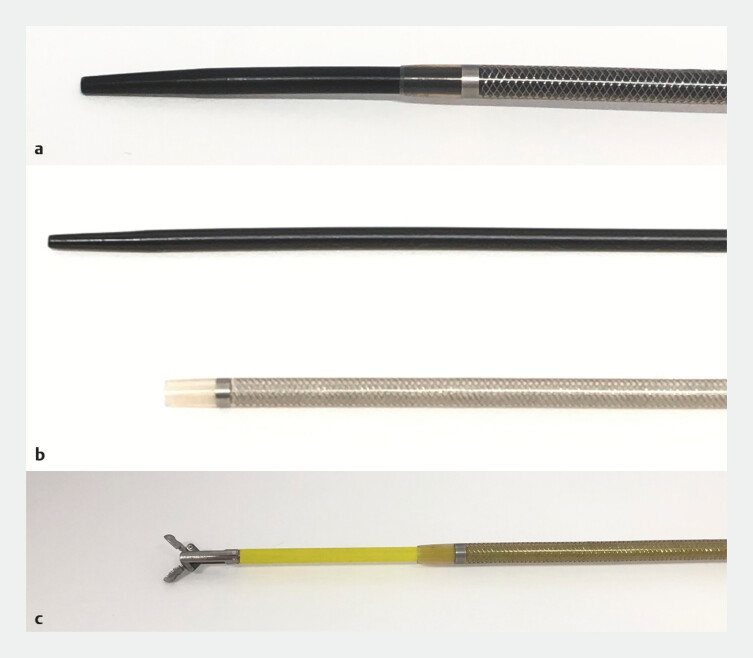
An overview of the tapered guiding sheath system (EndoSheather, Piolax, Kanagawa, Japan) and its structural features. **a** A close-up view of the distal end of the system. The inner catheter tip is tapered (arrow). The outer diameter is continuous at the junction between the inner catheter and the outer sheath, with no step-off (arrowhead). **b** The distal end of the outer sheath after the removal of the inner catheter (white arrow). A radiopaque marker is located near the sheath tip (black arrow), facilitating fluoroscopic visualization. **c** The distal end after the insertion of biopsy forceps through the outer sheath, demonstrating its role as a delivery conduit for accessory devices.

Preoperative biliary drainage for perihilar cholangiocarcinoma is technically demanding, and unexpected device-related failure may jeopardize timely decompression. The EndoSheather (Piolax, Kanagawa, Japan) is a 7.2-Fr tapered-tip guiding sheath that, upon removal of its inner catheter, can serve as a low-kink conduit for devices up to approximately 6 Fr ( Fig. 1 ) 1 2 .

An 82-year-old woman presented with obstructive jaundice due to perihilar cholangiocarcinoma. Endoscopic retrograde cholangiopancreatography (ERCP) was performed for preoperative biliary drainage. The placement of a fully covered self-expandable metal stent (FcSEMS; HANAROSTENT, 6 mm × 120 mm; Boston Scientific, MA, USA) was attempted for surgical bridging. Although the delivery system reached the bile duct, the outer sheath could not be retracted, preventing stent release. Intraductal angulation and kinking of the delivery system were suspected to have impeded withdrawal.

**Fig. 2:**
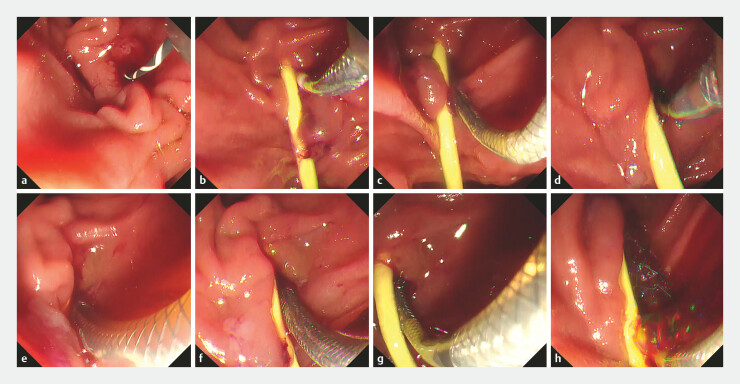
Endoscopic views of guiding-sheath–assisted rescue metal stent deployment. **a** Attempted transpapillary biliary drainage for perihilar cholangiocarcinoma using a metal stent delivery system. **b** Release failure: although the delivery system advanced successfully into the bile duct, the outer sheath could not be withdrawn and the metal stent could not be deployed. **c** The delivery system was partially deployable outside the patient, suggesting intraductal angulation/kinking as the cause of release failure. **d–h** The salvage technique using a tapered-tip guiding sheath (EndoSheather). The guiding sheath was positioned at the target site **d** , the inner catheter and the guidewire were removed **e** , the metal stent delivery system was inserted through the outer sheath and partially deployed (~1/4) within the sheath **f** , the sheath was gently pulled back to fix the stent tip at the intended position **g** , and the final deployment was completed while gradually withdrawing the sheath to maintain the accurate placement **h** .

**Fig. 3:**
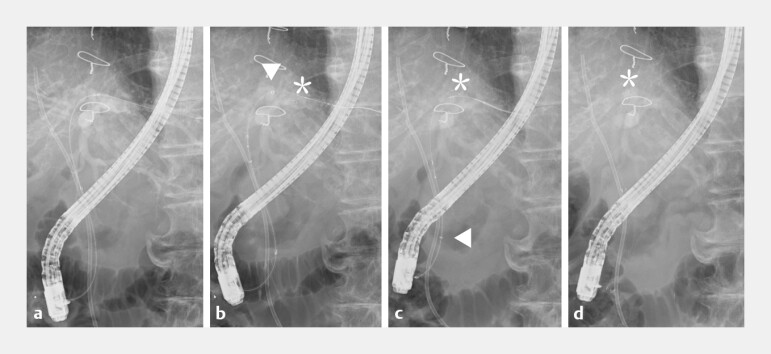
Fluoroscopic views of the rescue deployment technique using a guiding sheath. **a** A plastic stent was placed successfully; however, metal stent release failed because the outer sheath could not be retracted within the bile duct. **b** After the partial deployment of the metal stent within the guiding sheath, the assembly was advanced to the target position (arrowhead, sheath tip; asterisk, stent tip). **c** The sheath was pulled back to accurately fix the stent tip (arrowhead, sheath tip; asterisk, stent tip). **d** Final deployment was completed while withdrawing the sheath, achieving precise stent positioning (asterisk, stent tip).

As a salvage strategy, the EndoSheather was used as an “external guiding sheath” for controlled stent release. The sheath was advanced to the deployment site, and the inner catheter and the guidewire were removed. The FcSEMS delivery system was then inserted through the outer sheath. Before deep advancement, the stent was partially deployed (approximately one-quarter) within the outer sheath to create a stable leading segment. Under fluoroscopic guidance, the assembly was advanced to the target position. The outer sheath was then gently retracted to fix the stent tip, and full deployment was completed while avoiding proximal displacement ( Fig. 2 and Fig. 3 , Video 1 ).

Guiding-sheath-assisted rescue deployment of a fully covered biliary metal stent after release failure during endoscopic retrograde cholangiopancreatography.Video 1

This guiding-sheath-assisted rescue technique, originally developed as a dedicated delivery conduit and later adapted for drainage stenting 1 2 3 , provides additional pushability, kink resistance, and positional control when conventional transpapillary stent release fails. By creating a coaxial, kink-resistant conduit with a radiopaque distal marker, the guiding sheath helps reduce device bending, improve pushability, and enable incremental, fluoroscopy-controlled deployment to achieve accurate stent positioning.

Endoscopy_UCTN_Code_TTT_1AR_2AZ
